# Researchers, animal support and regulatory staff: symbiosis or antagonism?

**DOI:** 10.1186/s42826-022-00129-0

**Published:** 2022-07-08

**Authors:** Benjamin Tsang, Robert Gerlai

**Affiliations:** 1 Department of Psychology, University of Toronto Mississauga, Toronto, Canada; 2 Department of Critical Care Medicine, Hospital for Sick Children, Toronto, Canada; 3 Department of Cell and Systems Biology, University of Toronto, Toronto, Canada

**Keywords:** Animal welfare, Vivarium, Animal user, Animal support staff, Regulatory staff, Compliance, Animal models

## Abstract

Animals are studied en masse by biologists around the world in a variety of biomedical and basic research studies. All this research benefits humankind and animals alike as it tackles a wide variety of problems ranging from those of conservation biology to medicine. Research with animal subjects is a complex endeavor that requires the cooperation and collaboration of a large number of experts, from the principal investigator through technicians and vivarium staff to regulatory experts. The research must be conducted in a humane manner that adheres to acceptable practices regulated by local, state and federal guidelines, rules and the law. In this short opinion article, we examine the current state of affairs regarding how researchers, animal support staff and regulatory experts work together. We pay particular attention to potential conflicts that may arise from the occasionally distinct roles played by those involved in animal research, and we provide some suggestions as short- and long-term remedies that have not been previously discussed in the literature.

## Background

Increasing administrative bureaucracy and animal welfare requirements is becoming more common in all facets surrounding the use of animals for research. However, to date, there has been little to no recommendations or solutions to these ever-increasing complexities, which have left animal users, animal care staff, and veterinary teams in often conflicting situations.

## Main text

Research is a complex endeavor. It may have a medical orientation, i.e., it may on the short- or the long-run lead to better understanding of a human or animal disease and/or the development of treatment or cure for the disease. It may also be about increasing our general knowledge, understanding of the living world, which then, indirectly though, will also improve quality of life for us, humans, and for all our non-human fellow animals. Both types of research are crucial, yet occasionally challenged with regard to their legitimacy or appropriateness by some activists who may feel animal research is unethical. Scientists of both biomedical and basic research-oriented studies agree that appropriate research can only be accomplished if the research subjects, animals in this case, are kept under optimal conditions, and that they must be healthy and treated well. While some research may require invasive approaches, procedures that may be unpleasant or harmful to the animal, scientists also agree that research on animals must be conducted in a humane manner. No animal should suffer, and if an invasive procedure is required, there must be a very good reason for choosing it. In other words, all alternatives must be considered first, and suffering must be minimized or completely eliminated if possible.

But how do we ascertain that this is actually accomplished? Can we trust the scientists, animal technicians, students, all those who conduct the research and actually work with the animals? In our several decades long scientific career, our experience has been that the answer to this question is yes. Nevertheless, it is still crucial to exert oversight, some control over the research scientist. He/she may not be aware of all regulations, rules relevant to his/her research, and/or he/she may not have considered all alternatives, possible solutions that could lead to the most humane use of animals in his/her research. Regulatory staff could provide such advice and required oversight. Regulatory and compliance monitoring procedures and the roles of committees and representatives involved in such procedures have been well described in the literature [1, 2].

In the variety of academic, biopharmaceutical and biotechnology research laboratories in which we have worked, we also noticed that research staff often have to rely upon the expertise of the vivarium animal support staff, animal technicians and veterinarians, who are knowledgeable about the maintenance, feeding, breeding, general care and healthcare of animals. This is also an important component of research. Most scientists appreciate that without healthy, optimally maintained animals, appropriate research results cannot be obtained. Thus, biomedical and basic research requires coordinated cooperation among all three groups of experts, research personnel, regulatory experts and animal support staff. However, representatives of these three pillars of research may not always work together seamlessly as they play different roles from which conflicts and misunderstandings can arise. In this short opinion article, we explore a few examples of such conflicts, briefly discuss the underlying issues, and try to provide short-term as well as long-term solutions.

### The animal support staff and their role in research

Animal support staff provide a range of services that cater to the needs of the studied animal species, and thus help research staff, scientists, students and research technicians who would like to conduct experiments with them. In most animal facilities, usual animal support staff duties include housing maintenance, changing and cleaning cages or enclosures, food replenishment, and animal welfare and health related quality checks and procedures. Without animal support staff, animal research would likely grind to a halt for most researchers. The specific husbandry experience of animal support staff and their routine schedule are invaluable, especially to those researchers who are adopting a new species or to those who are less familiar with the needs and species-specific features of their animal subjects. However, recently yet another duty has been relegated to animal support staff: compliance monitoring. New regulatory guidelines often require increasingly bureaucratic health monitoring, e.g., the creation of paper (and/or electronic) trail, documenting compliance of research staff with rules and regulations. This novel role for the animal support staff, as important as it may be, is often perceived by researchers as policing their work, and may create an adversary relationship. In simple terms, researchers may feel that people “who don’t understand their work” are looking over their shoulder, and animal support staff may feel that researchers are “cutting corners and are not listening”. In some cases, the conflict between animal support staff and researchers may deteriorate to a “gothca-culture”, potentially ruining the crucial and mutually beneficial cooperation between animal support staff and the researcher. The fundamental issue, to which we will return in a more general sense later, is the unidirectionality of the relationship between animal support staff and the researcher. The former has authority and the latter has to comply, but mechanisms that would allow bidirectional discussions on what is appropriate, what steps may best remedy perceived or real issues, are often lacking. Consider the below real-life example we drew from zebrafish research.

### Rigid guidelines versus decades of experience: A zebrafish example

One way compliance may be achieved and monitored most efficiently is if appropriate guidelines and rules are designed for how to use animals in research. Such guidelines are intended to delineate procedures and actions the researcher must perform if a specific issue arises. But research is varied, and animal health and welfare problems are more complex than what rules, regulations, guidelines may be able to foresee or tackle. How can animal support staff and researchers address unforeseen or novel issues? Here, we use zebrafish, a relative newcomer in biology research, to exemplify the problem.

Zebrafish are housed in high density system racks that automatically perform numerous animal husbandry related procedures, including mechanical, chemical and biological filtration, salinity (salt concentration) control as well as water changes. This level of automation has largely relegated animal support staff to the aforementioned compliance monitoring roles in most zebrafish facilities. Despite the training animal support staff receive on the particular study species, zebrafish in this case, the required on-line modules and hands-on demonstrations do not replace decades of aquaculture experience. For example, an animal technician may be taught that a pH range between 6.5 and 7.5 is ideal for zebrafish. In principle, this is correct as this is indeed the mid-range of pH values zebrafish may experience in their natural habitat [3], and this is also the range most zebrafish facilities employ [4–6]. Once the pH recorded from the zebrafish tank is found outside this range, the recommended corrective measure is to raise it by adding sodium bicarbonate (baking soda). Thus, if the researcher does not respond immediately, say, he/she does not raise pH from the out-of-range value of pH 5.5 back to 7.0 (neutral), non-compliance is noted, the researcher is warned and must take immediate corrective steps. From the perspective of animal support staff, artificially raising the pH back to the optimal range is the required action, and once achieved and documented, the issue of non-compliance has been deemed resolved. However, there are two fundamental issues with this. One, rapid change from pH 5.5 to 7.0 is more harmful to zebrafish than keeping the fish at a pH that is slightly out of range. Two, low pH in aquaculture systems, including the often-employed high-density zebrafish rack systems, is the result, and not the cause, of problems. It is the result of accumulation of excess amounts of organic waste, resulting from poor filtration, overfeeding, overcrowding, or the combination of these factors. Raising pH to meet the required pH range would only mask the underlying issue. One must remedy the root cause, and e.g., reduce the amount of delivered food, decrease stocking density, and/or fix/improve biological filtration.

Could all the pieces of information described in the above example be added to the description of required corrective procedures and guidelines on how to keep zebrafish? Yes, of course. Could all variations of specific problems and contribution of unique combinations of factors and circumstances to such problems be generally tackled by such guidelines? Naturally, no. What is the solution then? First, let us unequivocally state that the issue is not that animal technicians, animal support staff or researchers using animals are not knowledgeable enough about animal husbandry, maintenance, health and welfare, or that one group is better than the other. The issue is that each member of this team may have incomplete knowledge. Thus, only open, collegial dialogs, discussions, and mutual respect for the talents, backgrounds and experiences of the team members can lead to the optimal solution. We deliberately call the group of people involved a ‘team’. But this is exactly what gets lost in the hierarchical manner in which animal support staff and research personnel are currently organized at most Universities, Academic Research Institutes or Industry animal research facilities. Animal support staff tell the researcher what and how to do. Communication is essentially unidirectional. Of course, in most animal facilities and research studies, people develop good work relationships and appreciate the importance of bidirectional communication. Nevertheless, the current design of the system works against this respectful collaborative spirit. The solution for this problem we will return below.

### Is increased bureaucracy good or necessary?

We have alluded to this above: one of the most concerning and pervasive issues to date is the disconnect between placing substantial bureaucratic burden on research staff and believing that rigid rules, check-sheets, paper trails, hiring an ever-increasing number of compliance officers into regulatory positions and strengthening research oversight are the solution. The main issue here is that bureaucracy is like an organism that is evolving in one direction and one direction only: increasing complexity. This “cultural evolution” has led to numerous newly added layers in how we deal with animal health and welfare issues. Compared to the past, we now must complete larger number of check-sheets, we have more boxes to tick, and we have implemented increased oversight. Two decades ago, there was much less bureaucracy. Animal support staff and research staff often had more time to attend to the animals and develop insightful discussions with each other. Does our currently bloated bureaucratic system really better serve the animals employed in research? We do not think so. The bureaucratic process creates an illusion. It makes people believe that things are taken care of, and it also makes the user feel less responsible for taking action. We all have become part of a giant autonomous conveyor belt. Being a tiny component of this large machine takes away initiative and personal responsibility. Increasing bureaucracy and tightened compliance monitoring, we argue, may accomplish the opposite of what it was intended to do.

### Do we need to solve anything? Ideas for the short run

Do we need to change anything? Do the above exemplified issues really represent problems? Is the system, as it is set up currently, broken? One can argue that the answer to these questions is no, the system is actually working. If one looks at overall research output, i.e., the annual number of scientific papers published, the number of discoveries made per year, and the rapidly advancing fields of biology, biomedical research and basic research alike, one can easily argue that nothing is wrong. We are witnessing exponential growth both in quantity and depth of biology research. Our research facilities are well maintained, and our animals are healthy. But could we do better? We argue that the rapid advancement of knowledge in animal research is achieved not because but despite the current system, despite the problems plaguing the way our animal support staff, regulatory experts and research staff work together.

Others may argue that the above exemplified problems represent the limited experience of the authors of this paper, or perhaps are unique to a small number of specific laboratories, research institutes or universities, and do not represent general trends across the world. However, even a brief survey of the literature suggests otherwise. For example, Abbott [7] reports on data and conclusions published by the European Commission regarding the use of animals in scientific research, and cites the head of the German Primate Center as saying “complex reporting requirements put a high administrative burden on scientists and their organizations”. The same author mentions the conflict between what researchers must endure and what the European Commission regards as reasonable. Kwon [8] reports that “Swiss researchers struggle to get animal experiments approved” due to the increasingly burdensome, arbitrary and complicated bureaucratic animal research approval process. The situation is not better in North America either, as increasing regulatory burden is a major issue here too [9–11]. Thulin et al. [12], for example, discuss elevated costs resulting from the ever-growing regulatory burden as well as the “overly complex compliance organizations and unnecessary policies and procedures” in the USA. Discussing similar issues, Haywood and Greene [13] conclude that “overzealous application of “requirements” does not necessarily benefit the animals”, and suggest that “clear and consistent communication among all stakeholders—the institutional leadership, institutional animal care and use committee (IACUC), attending veterinarian and staff, and scientists” is needed to solve the issues. Cornwall [14] in his news-piece cites a report released by the U.S. Department of Agriculture, National Institutes of Health and the Association of American Medical Colleges calling for simplified regulations and giving “researchers increased say in crafting new rules”. In fact, the problems have been becoming so acute that even workshops have been organized to discuss how to reform animal research regulations and reduce regulatory burden [15]. These problems seriously affect quality of work with the animals but also importantly the quality of life of those working with the animals. Partly due to having to navigate the complexities of research with animals, mental well-being of laboratory animal professionals is declining [16–18], a major issue almost never considered in the context of regulating animal research.

What is the solution? Every University, Research Institute, Laboratory has some unique local issues. A general, overarching advice may not work. But reviewing the literature and based upon our own decades long experience in a variety of Universities and Industry research facilities across the Globe suggest that perhaps there are some common elements in the problems in most places. These common problems one may be able to tackle on the short-run in a similar manner. One of the problems is communication, as Haywood and Greene [13] emphasized. Development of proper bidirectional communication and showing respect among the team of the three groups of people (animal support staff, regulatory experts and research staff) would go a long way. How to implement this, of course, depends upon substantial good will and personal initiatives at the grass roots level. The second element is bureaucracy. Our experience is that it has been steadily increasing in most places. Yet, it should be trivial that reduced bureaucracy is better for everyone. Fewer boxes to tick by research staff requires fewer tick-marks to check by animal support staff and fewer potential issues to deal with for compliance officers, and more time for researchers and animal support staff to actually work on the often idiosyncratic and no-repeating issues that pop up during the maintenance and research with the animals. But how does one go about addressing all this on the long run?

### Long term solution: build negative feedback loops (breaks) into the system

The natural tendency is to try to address every possible combination of factors/problems, develop more guidelines, checks and oversight, i.e. to increase bureaucracy. Once a decision to include an “important” end point, a “crucial” question about health monitoring, or the use of an SOP (standard operating procedures) has been made and added to the system, these requirements/points remain in the system. Subsequent modifications almost always include these added complexities, upon which further additions are then based as the system “develops” towards increased complexity. Similarly, once a regulatory position has been created, or once an administrative role has been deemed necessary and the positions are filled, they generate requirements for further support staff, assistants, organizers and heads of organizers. Furthermore, once these different arms of staff members have been created, a bit of instinctive territoriality (human species-specific feature) may kick in: the view of ‘them versus us’ may develop and thus created abstract territories are defended. In sum, just like in biological evolution [19], it is much easier to add to existing system features than to remove from them. The above exemplified tendency towards increasing complexity is not unique to how our animal research related systems evolve. It is typical of cultural evolution in general, and can be seen in practically every aspect of our society: we all expect growth, bigger and more. Can we reverse this natural tendency?

The long-term solution is quite simple in principle, but difficult in practice. We first consider the principle. Any organism, organization, and for that matter all systems in general, will go out of control without negative feedback loops. Right now, the way regulatory aspects of animal research are set up, the way regulatory experts, animal support staff and research staff work with each other, represent a system without feedback loops. The research staff is dependent upon the animal support staff and is also at the mercy of compliance officers and regulatory staff. But these latter staff members do not depend on research staff. The situation is akin to a car that only has an accelerator, but no breaks. If the animal support staff demands some action, the researcher has no choice but to comply and do as told. If a regulatory agency decides to bring new guidelines, animal support staff must implement them and make research staff comply. Information, and resulting consequences (what we call direction of causality), flows one way: regulatory agency to animal support staff to research staff.

The worst example of what this can do, which frankly devastated the zebrafish research landscape in Canada, is how and why the Canadian Food Inspection Agency decided to regulate importation of zebrafish to that country [20]. The practical solution to this unidirectional information flow-causality principle is difficult, perhaps even painful, and could only be accomplished on the long-run: establishment of efficient negative feedback loops in the system. Good will, proper communication, common sense may not be enough. What could represent an efficient feedback loop?

First consider an example for an efficient feed-forward loop, i.e., how research staff are compelled to comply. This will illuminate what it would take to develop a break in the system. When a non-compliance is detected, the researcher in most research facilities is sent a message, an automated or personalized warning about the non-compliance issue. This message may explain that unless the problem is remedied, the researcher’s animals will be transferred and funding for his/her research will be suspended. Such events, if they occur, may have serious consequences, as the researcher will not be able to conduct his/her work, and lack of publications leading to lack of future grant funding obtained, reduction in number of students etc., may all be factored into the researcher’s annual performance evaluation. Thus, a non-compliance related warning is serious and will motivate the researcher to make sure the problem is addressed fully and promptly. This is an efficient feed forward mechanism. Feed forward in the sense that the information flow goes the way we described above. What would represent an efficient feed-back mechanism, reversal of the direction of causality? Assume that the regulatory rule set by a Government Agency is incorrect, like the requirements set by the Canadian Food Inspection Agency for the importation of zebrafish into Canada. These requirements, arguably, were based upon a faulty interpretation of information published in a peer reviewed journal, among other issues [21]. Plenty of evidence was provided to the Agency, including scientific evidence, along with numerous practical as well as theoretical arguments against the Agency’s decision [20]. The Canadian zebrafish research community unanimously rejected the Agency’s decision for the debilitating importation requirements. Furthermore, numerous University officials also contacted the Agency, asking them to reverse their decision. However, the Agency did not budge. Why? Because it had little to no incentive to do so. Staff at the Agency were not dependent upon the zebrafish research community, or how their decisions are viewed by experts and University officials alike. Their performance evaluation did not factor in the feedback. Their salaries and livelihood remained unaffected. Similar issues abound at lower levels, at a smaller scale in the system. Just as animal support staff and University representatives could not effectively persuade the Government agency to change its decision, research staff also often have a hard time influencing University administrators, Veterinarians or other animal support staff about correcting erroneous rules/regulations decisions. The long-term solution can only be proper incentivization of all team members. In other words, performance of all participants, including Government Agency employees, University officials, animal support staff, and not just that of research staff, must be made dependent on feedback. Proper mechanisms for feedback from the lower to the higher level, i.e., reversing the direction of information flow (research staff to animal support staff to regulatory agency) and having measurable consequence/effects of the feedback provided should be established.

## Conclusions

Short term solutions (months to few years). We need to harness the good will and collegial nature of all participants in animal research and facilitate communication among research and animal support staff and regulatory experts. We must promote education of research staff about regulatory requirements as well as about the role and daily activities and responsibilities of animal support staff. We should encourage animal support staff to learn about research conducted by research staff, ask animal support staff to attend in house research presentations by students and principal investigators, invite them to laboratory meetings, departmental events, and overall involve them in the research process as much as possible. We should also encourage animal support staff, veterinarians and University officials alike to be critical, and evaluate the correct nature, of government or regulatory agencies’ decisions about rules, regulations and guidelines. We should insist on establishing mechanisms via which feedback may be sent to the regulatory agencies involved. Long term solutions (years to decades). We must attempt to create an incentivization structure, mechanisms via which the above short-term goals may be achieved. We will need to think about both positive (reward) and negative (punishment) based incentives developed not only for research staff but also for animal support staff and administrators and decision makers at regulatory agencies. We will need to establish mechanisms that would not only facilitate but would mandate appropriate feedback, feedback that will represent backward flow of information as well as meaningful effect, i.e. consequences, of this information flow. We must achieve reversal of the direction of causality and create a system where research staff can evaluate the quality of work provided by animal support staff and by Government agencies.

## Data Availability

Not applicable.

## References

[1] Mohan S, Huneke R (2019). The role of IACUCs in responsible animal research. ILAR J.

[2] Hanwell D, Gerlai R (2018). Ethical considerations for animal use in behavioral and neural research. Molecular-genetic and statistical techniques for behavioral and neural research.

[3] Parichy DM, Postlethwait JH, Gerlai R (2020). The biotic and abiotic environment of zebrafish. Behavioral and neural genetics of zebrafish.

[4] Tsang B, Zahid H, Ansari R, Lee RC, Partap A, Gerlai R (2017). Breeding zebrafish: a review of different methods and a discussion on standardization. Zebrafish.

[5] Tsang B, Ansari R, Gerlai R, Gerlai R (2020). Maintenance and breeding of zebrafish, with some ethological and ecological considerations in mind. Behavioral and neural genetics of zebrafish.

[6] Tsang B, Gerlai R, D’Angelo L, de Girolamo P (2021). Breeding and larviculture of zebrafish (*Danio rerio*). Laboratory fish in biomedical research.

[7] Abbott A (2020). Animal-research data show effects of EU’s tough regulations. Nature.

[8] Kwon D. Swiss researchers struggle to get animal experiments approved. In: The Scientist. 2019. https://www.the-scientist.com/news-opinion/swiss-researchers-struggle-to-get-animal-experiments-approved--65293. Accessed 15 May 2022.

[9] Pritt S, McNulty JA, Greene M, Light S, Brown M (2016). Decreasing institutionally imposed regulatory burden for animal research. Lab Anim (NY).

[10] Norton JN, Reynolds RP, Chan C, Valdivia RH, Staats HF (2017). Assessing the satisfaction and burden within an academic animal care and use program. FASEB J.

[11] Pohl AD, Wallace RG (2017). Response to protocol review scenario: let's talk about self-imposed regulatory burden. Lab Anim (NY).

[12] Thulin JD, Bradfield JF, Bergdall VK, Conour LA, Grady AW, Hickman DL (2014). The cost of self-imposed regulatory burden in animal research. FASEB J.

[13] Haywood JR, Greene M (2008). Avoiding an overzealous approach: a perspective on regulatory burden. ILAR J.

[14] Cornwall W (2017). United States should dramatically retool animal research rules, groups say. Science.

[15] Reforming Animal Research Regulations. Workshop recommendations to reduce regulatory burden. 2017. https://www.aamc.org/media/12231/download?attachment. Accessed 15 May 2022.

[16] Randall MS, Moody CM, Turner PV (2021). Mental wellbeing in laboratory animal professionals: a cross-sectional study of compassion fatigue, contributing factors, and coping mechanisms. J Am Assoc Lab Anim Sci.

[17] Murray J, Bauer C, Vilminot N, Turner PV (2020). Strengthening workplace well-being in research animal facilities. Front Vet Sci.

[18] LaFollette MR, Riley MC, Cloutier S, Brady CM, O'Haire ME, Gaskill BN (2020). Laboratory animal welfare meets human welfare: a cross-sectional study of professional quality of life, including compassion fatigue in laboratory animal personnel. Front Vet Sci.

[19] Gerlai R (2020). Evolutionary conservation, translational relevance and cognitive function: the future of zebrafish in behavioral neuroscience. Neurosci Biobehav Rev.

[20] Hanwell D, Hutchinson SA, Collymore C, Bruce AE, Louis R, Ghalami A (2016). Restrictions on the importation of zebrafish into Canada associated with spring viremia of carp virus. Zebrafish.

[21] Sanders GE, Batts WN, Winton JR (2003). Susceptibility of zebrafish (*Danio rerio*) to a model pathogen, spring viremia of carp virus. Comp Med.

